# Why rare disease needs precision medicine—and precision medicine needs rare disease

**DOI:** 10.1016/j.xcrm.2022.100530

**Published:** 2022-02-15

**Authors:** Matthew Might, Andrew B. Crouse

**Affiliations:** 1 Hugh Kaul Precision Medicine Institute, University of Alabama at Birmingham, Birmingham, AL, USA

## Abstract

With one in ten suffering from one of 10,000 rare diseases, precision medicine opens a path toward identifying therapies for rare patients. Conversely, it is rare patients—through their collective experience and the knowledge captured in their genetics—who open the path toward identifying therapies for common patients.

## Main text

For much of human history, patients suffering from rare diseases have found themselves beyond hope. With an estimated one in ten suffering from one of roughly 10,000 known rare diseases,^1^ that is an extraordinary scale of suffering to endure in the shadow of hopelessness.

(In the context of this article, the term *rare disease* will mean *rare genetic disease*, given that rare genetic diseases compose the majority of rare disorders. We discuss the incorporation of non-genetic rare diseases—such as autoimmune and infectious diseases—in the broader strategy we outline toward the end.)

For many patients, merely diagnosing the rare disease that afflicts them can be a sobering challenge.^2^ And, even after the diagnosis, there is a jarring lack of treatments available for nearly all known rare disorders.

To be certain, rare diseases remain a challenge to diagnose and treat even today, yet with the emergence of precision medicine, there is reason to believe that this challenge is not as insurmountable as it once was.

Precision medicine in the most general sense means tailoring treatment directly to the specific characteristics of an individual patient.^3^ It is about understanding a patient’s condition with as much resolution (and data) as possible and attempting to identify chains of causes that lead all the way back to a root molecular cause.

As it turns out, if we are to achieve the promise of precision medicine for all diseases—rare and common alike—then there is sound reasoning for society as a whole to apply precision medicine to and learn from rare diseases.

### The shifting landscape for rare diseases

Scientific progress in areas like genomic sequencing has shifted the capabilities of medicine in the diagnosis of rare diseases, leading to a surge in discoveries of novel (and often ultra-rare) diseases. At the same time, science itself is further shifting its own capabilities in terms of being able to efficiently study rare diseases. CRISPR, as one example, has dramatically reduced the time and cost of producing a genetically modified organism that represents a disease, or even a specific patient.

It is the fusion of these two shifts—in diagnostics and in genomic sciences—that first made precision medicine tractable. This fusion has substantially transformed what is feasible for individual patients (or small numbers of them). And, with many individual rare disease patients pressing the science forward, their collective efforts have outlined the contours of an emerging “algorithm” for precision medicine—a playbook for patients to follow as they navigate both diagnostic and therapeutic odysseys in a structured, step-by-step fashion.

Less noticeably, that same fusion has realigned incentives for society as whole with respect to rare disease as well. This realignment leads to compelling scientific, economic, medical, and moral reasons to invest in studying rare diseases. Just as importantly, the same fusion provides a roadmap for *how* to make these investments. In particular, large-scale study of rare disorders promises to substantially accelerate the *semantic* mapping of the human genome as well as subsequent pharmacological mastery of it.

### The paradox of precision medicine

Paradoxically, precision medicine is *easier* for rare diseases.

This relative ease is in part because with rare genetic disorders, the root molecular cause comes almost immediately with the diagnosis itself: a pathogenic genotype driving disease *is* exactly the kind of root cause that precision medicine aims to identify in every patient, rare or common.

While finding the root molecular cause of harm *can* be done with common diseases, it is not always straightforward, and especially so in the context of an individual patient. There is no equivalent of genomic sequencing in the common disease space; there is no technique available to so reliably draw a “molecular bullseye” right on the underlying cause of a given patient’s condition.

Moreover, unlike those of common disorders, this root cause may be the only mechanism one needs to correct to achieve relief. In contrast, patients with the same “common” disease may have illnesses that stem from a variety of interacting mechanisms—mechanisms that may differ from patient to patient, even when they suffer from the “same” disease.

Consider, for a moment, diabetes. At a high level, the only molecular trait in common between all diabetics is hyperglycemia. However, the mechanisms behind hyperglycemia between any two patients are likely to be different. While in some cases the root molecular driver of that hyperglycemia is known for an individual diabetic, in others, it is not. In the context of diabetes, it is the ambition of precision medicine to know the root cause of hyperglycemia for every individual diabetic—and to tailor treatment to that root cause or a mechanism downstream of it.

Thus, rare disease has an extraordinary advantage in precision medicine: genomic sequencing jumps many patients with rare diseases directly to the next phase of precision medicine—precision therapeutics—where addressing the root cause with a targeted therapy is the goal.

Because of this head start on knowing the root cause, individual rare disease patients have begun to map out exactly what the process of identifying targeted therapies looks like.

### The algorithm for precision medicine

Both as the father of a child who passed away from an ultra-rare genetic disease (NGLY1 deficiency) and now as the Director of the Hugh Kaul Precision Medicine Institute at the University of Alabama at Birmingham, I can offer both a first-person and high-level view of the emergence of both precision diagnostics and precision therapeutics.^4^

Having spent nearly a decade searching for treatments for my son after his diagnosis (and even finding some that benefited him in his lifetime), I have turned my professional attention in the last 5 years toward generalizing the process of identifying targeted therapeutics for individual patients—toward a more general “algorithm” for precision medicine.^5^

While my son’s diagnostic and therapeutic odysseys^6^^,^^7^ provided inspiration and scaffolding for this algorithm, my perspective as a guide and partner to many patients and parents in their own search for diagnoses and therapies has provided a hill-top perspective from which to see this patient-derived algorithm take shape.

Remarkably, the algorithm, despite being developed largely in the context of rare genetic diseases, has significant implications for the remainder of medicine. At the moment, it is most directly applicable to fields that have molecular diagnoses for their patients. Oncology, for example, which has also benefited from the arrival of genomic sequencing, can readily adapt many of the lessons from efforts in precision therapeutics for rare diseases.

Without going into formal detail, at a high level, this emergent algorithm for precision therapeutics takes as input a molecular cause (such as a pathogenic genotype or an overactive pathway), and it outputs potential therapies that may ameliorate this particular cause—or it presents options for research that take a step toward illuminating those potential therapies.

To be clear, a compound identified by the algorithm as a potential therapy may not be a suitable therapy due to safety issues, an effective concentration too high for dosing in humans, or bioavailability in critical target tissues or cells. However, if it satisfies these concerns and is already approved, then single-patient, off-label usage is possible.

At the start, there’s a fork between two approaches: the rational and the empirical. Yet, the algorithm is non-deterministic in the sense that there is no need to take only one of these paths. However, with finite resources always a concern, there are often constraints that make one path more feasible or desirable than the other.

### The empirical path

With the empirical path, the molecular cause is transformed into a “precision disease model” of the patient’s condition. That model could be a cellular model taken from the patient; a cell line genetically or chemically modified to represent the patient; or a genetically tailored model organism such as a mouse, a fruit fly, a zebrafish, a worm, or yeast.

That model may make further study of the disorder tractable in ways that simply were not in the context of a single patient. In particular, some model organisms are nimble enough to be amenable to drug-repurposing screens, in which many drugs, or even all approved drugs, are tested for rescue of the condition.

As one remarkable and recent example of the empirical approach, the family of a child with PMM2-CDG commissioned such a precision disease model and subsequent drug screen, identifying epalrestat in the process. Subsequent n = 1 clinical trials have been extraordinarily successful in treating this child, and they have since led to the approval of a much larger trial for others with the same condition.^8^

### The rational path

With the rational path, the goal is to understand the *impact* of the perturbed mechanism in the patient and to then invert it with a therapy. In general, a rare genetic disease may be said to have four kinds of impact with respect to the mechanism of harm: (1) the mechanism can become overactive; (2) it can become underactive; (3) it can become absent; or (4) it can acquire novel toxic behavior.

A growing number of methods address how to invert each kind of impact. Many of these methods are computational, and this means that, in some cases, predictions for what may help can come with little or no additional cost.

For instance, within NCATS at NIH, the Biomedical Data Translator^9^ is an ambitious program that seeks to structure all biomedical data as computable knowledge, make it eligible for automated reasoning via artificial intelligence, and then make it accessible enough to rapidly accelerate translation from bench to bedside.

Translator can look for previously undiscovered ways to alter the activity of given genes or mechanisms once they’ve been identified as integral to a patient’s condition.

For example, Translator can search for an inhibitor of a particular target gene—relevant when the impact is understood to be a gain of function.

In fact, there are signs of clinical success emerging from this approach, with the most recent being the clinical trial of a therapy—low dose ketamine—predicted by Translator for the rare autism spectrum disorder ADNP syndrome.^10^

Beyond the identification of small molecules, the rational approach can also suggest the design of genome-directed therapies, such as antisense oligonucleotides, gene-editing techniques, and gene therapies. In fact, the patient-driven development of a custom single-patient antisense oligonucleotide shows that, while not yet scalable to all patients, individualized genome-directed therapies have now become possible.^11^

### From rare to all

It is becoming clear that the methodology for precision medicine being developed within the rare disease community has broader implications for the rest of medicine. These empirical and rational approaches to finding therapies will ultimately apply to any disease (or patient) for whom the molecular causes are identifiable.

### The need for a Rare-Powered Precision Medicine Initiative

While society will certainly benefit from the pioneering rare disease patients carving out the algorithm for precision medicine—and would benefit from investing in their efforts to carve it—society can also benefit from the study of rare diseases more directly.

That benefit comes from the implicit understanding of the human genome locked away in rare disease patients. While many common conditions may not have a simple monogenic cause, they can often be traced back to individual molecular mechanisms, and each of *those* mechanisms *does* have an associated gene or network of genes.

Manipulating these molecular mechanisms is critical to precision medicine for common diseases, and that in turn reduces to understanding and manipulating the activity of genes—precisely the same problem being solved directly in precision medicine for rare disease.

The key difference is that precision diagnostics for common disease have not yet caught up to the resolution of genomic sequencing, but as the clinical era for transcriptomics, metabolomics, and proteomics nears, it is imaginable that we *will* find individualized molecular drivers for common conditions.

Cancers, if taken collectively as a “common” condition, have already experienced this shift, because in cancer, the same genomic diagnostics that work in rare genetic disorders already work for cancers as well. In fact, precision therapeutics in cancer differs in degree rather than in kind with respect to precision therapeutics in rare diseases.

The challenge at present is that a large fraction of the entire human genome remains poorly characterized *and* outside the reach of modern pharmaceuticals. Estimates of the “druggable” genome show that, at present, we have pharmacological influence over only about 3,000 of roughly 20,000 human genes.^12^

Fortunately, rare diseases offer an opportunity to accelerate our understanding and influence over much of the remainder of the human genome.

In much of biology, one of the best ways to understand the role of something is to remove it or perturb it and to observe what happens to an organism. In genetics, this approach is an essential tool to understanding what genes—and mutations to them—do. In fact, the Knockout Mouse Project^13^ has been systematically removing every gene from mice one by one and then deeply characterizing the result.

It is not ethical to remove a gene from a human being or mutate that gene in them and then watch what happens to get a better understanding of human genes. Fortunately for science—though tragically for patients—through naturally arising mutations distributed through the human population, many such “knockout human beings” do exist, and many of them are rare disease patients. This means that rare disease patients have collectively within them a potent source of knowledge about the human genome.

If we were to systematically study all rare disease patients, they could dramatically accelerate the construction and fidelity of a semantic mapping of the human genome—an index that maps every genetic element onto its meaning and function.

The Precision Medicine Initiative, launched by President Obama in 2015, made a bid to construct this mapping by enrolling a million Americans in a national research effort that would collect their health data as well as their genetics. A companion initiative, perhaps even within the structure of the original, that focused exclusively on rare disease patients could quickly provide high signal-to-noise data about the function and role of individual genes.

In fact, to a certain extent, a small-scale version of this experiment has already been running with the Undiagnosed Diseases Network (UDN), an NIH-funded national network of clinical sites in conjunction with a coordinating center and several cores for functional studies. Since its inception in 2014, the UDN has discovered many new diseases,^14^ illuminating the role of their corresponding genes within human biology for the first time.

Unfortunately, funding for the UDN is set to end soon, and as of yet, there is no equivalent effort to replace it, ending one of the most effective efforts there has ever been to accelerate understanding of the human genome—along with a significant national resource for alleviating human suffering.

### Addressing other rares: Autoimmune and infectious disease

While this commentary focuses on rare genetic diseases, it should be clear that other categories within rare disease, such as autoimmune and infectious diseases, will also benefit from the aforementioned investments in precision medicine and in a wide-scale rare-based Precision Medicine Initiative. Autoimmune diseases tend to have more accessible molecular mechanisms than other disorders and may too find themselves amenable to the approaches taken in precision medicine for rare disorders. And the treatments of infectious diseases (especially rare infectious diseases) are poised to benefit from the rapidly growing use of metagenomic sequencing to systematically catalog all potential pathogens inside a patient with a single test.

### Conclusion

Rare diseases have been given a reasonable purchase on hope with the advent of precision medicine. In fact, by simplifying some of the challenges within precision medicine, rare diseases have leapfrogged the rest of medicine in advancing practical applications of precision therapeutics, building infrastructure and processes for identifying targeted therapies that may ultimately benefit all patients.

At the same time, the systematic study of rare diseases presents a second opportunity for society as a whole to benefit from rare diseases by accelerating the *understanding* of the human genome through systematic study of all rare diseases.

Paired with the development of techniques for identifying targeted therapeutics, that understanding becomes the first step toward another major milestone: being able to pharmacologically influence the activity of any gene in the human genome.
